# Humoral Immune Responses in Dialysis Patients After mRNA Omicron JN.1 Vaccination

**DOI:** 10.1016/j.xkme.2025.101067

**Published:** 2025-07-07

**Authors:** Metodi V. Stankov, Markus Hoffmann, Christine Happle, Karsten Lürken, Amy Kempf, Inga Nehlmeier, Andrea Stölting, Stefan Pöhlmann, Alexandra Dopfer-Jablonka, Georg M.N. Behrens

**Affiliations:** 1 Department of Rheumatology and Immunology, Hannover Medical School, Hannover, Germany; 2 Infection Biology Unit, German Primate Center – Leibniz Institute for Primate Research, Göttingen, Germany; 3 Faculty of Biology and Psychology, Georg-August-University Göttingen, Germany; 4 Department of Pediatric Pulmonology, Allergology and Neonatology, Hannover Medical School, Hannover, Germany; 5 Dialysis Centre Eickenhof, Langenhagen, Germany; 6 Center for Individualized Infection Medicine (CiiM), Hannover, Germany

To the Editor:

Persons with end-stage kidney disease receiving hemodialysis are at high risk for severe coronavirus disease 2019 (COVID-19).^1^ Due to possible immune dysfunction, advanced age, and high prevalence of additional underlying conditions, including immunocompromising conditions, among individuals on hemodialysis, reduced vaccine effectiveness is a concern,^2^ and this holds true for infections with the severe acute respiratory syndrome coronavirus 2 (SARS-CoV-2) omicron variant.^3^ Although cohort studies suggest that updated COVID-19 vaccines from 2023/2024 provided protection against COVID-19-associated hospitalization, death, and thromboembolic events among patients receiving hemodialysis,^4^ preliminary data on the immune response to JN.1 mRNA vaccines in dialysis patients are lacking. We and others previously described robust immune responses to omicron-specific mRNA SARS-CoV-2 vaccinations in nephrology patients^5^^,^^6^ and recently demonstrated the impact of an updated, JN.1 omicron-adapted SARS-CoV-2 mRNA vaccine on neutralizing antibodies in healthy individuals.^7^ Here, we provide the first data on immune responses after mRNA omicron JN.1 vaccination in hemodialysis patients after multiple previous SARS-CoV-2 antigen exposures.

To assess JN.1 SARS-CoV-2 vaccination responses in hemodialysis patients, we analyzed changes in JN.1-reactive memory B cells (MBCs) and neutralizing antibody titers against contemporary omicron variants in 37 dialysis patients. The CoCo Study (German Clinical Trial Register DRKS00021152) and all analyses conducted for this article were approved by the Internal Review Board of Hannover Medical School (institutional review board no. 8973_BO_K_2020, last amendment Aug 2024). All study participants gave written informed consent and received no compensation. More detailed information on the methods employed can be found in Item S1 in the supplementary file. All hemodialysis patients received 30 μg of the updated mRNA omicron JN.1 vaccine (Bretovameran; BioNTech/Pfizer). The median age of patients was 68 years (range, 28-90 years), and 26 (70%) were male. Sixteen (43%) patients reported previous SARS-CoV-2 infections, and 37 (100%) had received previous vaccinations against SARS-CoV-2 (Table S1). All patients underwent hemodialysis, and the median duration of dialysis until JN.1 vaccination was 53 months (range, 4-289 months). The most frequent underlying kidney diseases were diabetic nephropathy (22%), followed by hypertensive kidney disease (19%) and IgA nephropathy (8%). Thirty percent of patients were obese, 32% had diabetes mellitus, and 62% reported underlying cardiovascular disease. Six (16%) patients received immunosuppressive medication. Four of them received treatment with oral corticosteroids (5 mg prednisolone in 3 and 20 mg hydrocortisone in 1), 1 patient received a combination of tacrolimus and everolimus, and 1 additional patient was under treatment with daratumumab.

First, we analyzed SARS-CoV-2 specific immunoglobulins (IgGs) in the plasma of vaccinated hemodialysis patients before and 21 days after vaccination. Median pre-vaccination anti-spike (anti-S) IgG antibody levels were 1,817.0 binding antibody units per milliliter (interquartile range [IQR], 2,670.9), and median anti-omicron S IgG levels were 170.4 relative units per milliliter (IQR, 266.1). Twenty-one days after vaccination, we observed a 3-fold change to a median of 5,413.0 binding antibody units per milliliter in anti-S IgG (IQR, 9,024.0), and a 4.7-fold change to a median of 796.0 relative units per milliliter in anti-S omicron IgG (IQR, 758.8; Fig 1A and Table S4).

**Figure 1:**
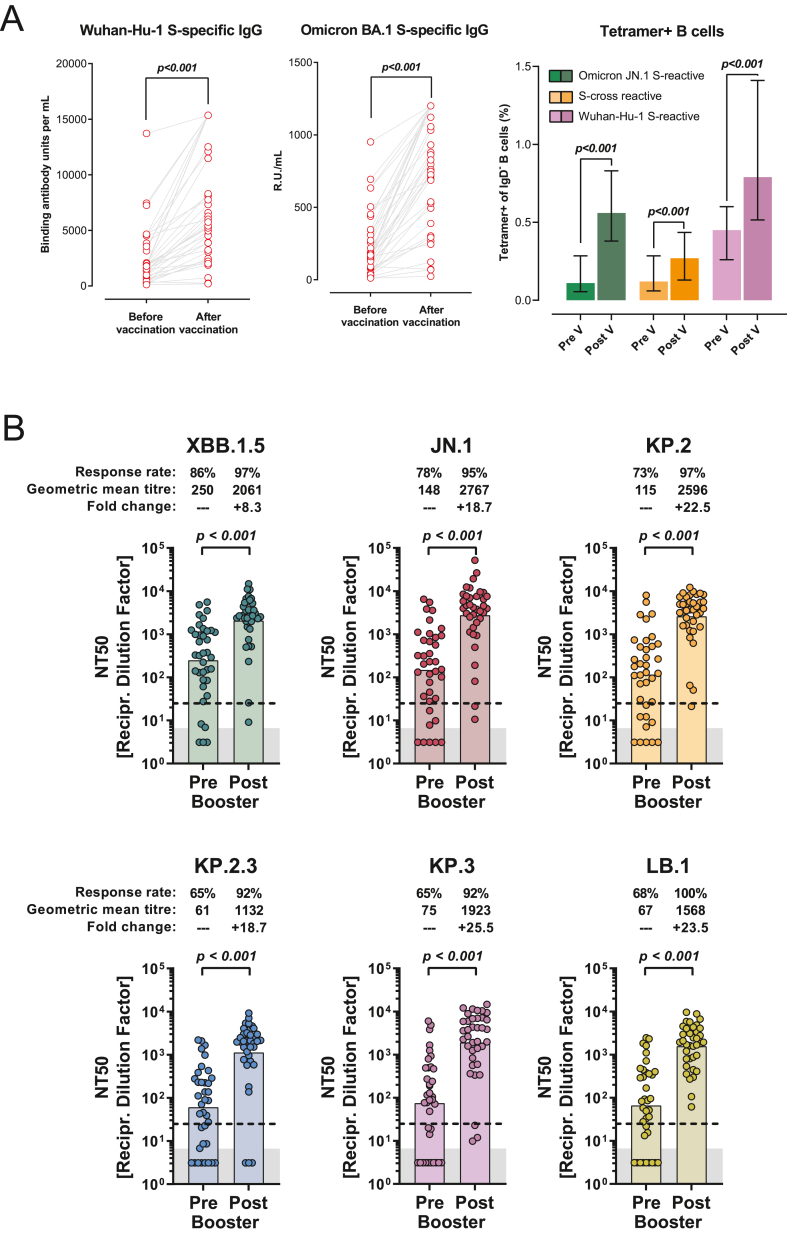
Humoral immune responses in hemodialysis patients following mRNA omicron JN.1 vaccination. (A) Concentrations of Wuhan-Hu-1 S-reactive IgG and omicron BA.1 S-reactive IgG in plasma (n = 37) obtained before or after vaccination with the mRNA omicron JN.1 vaccine (left and middle panels). Frequencies of memory B cells (n = 37) binding the receptor binding domain of JN.1 (green) to Wuhan-Hu-1 spike (pink) or both (cross-reactive, orange) before and after JN.1 vaccination (right panel). Data are represented as median (colored bars) and interquartile range (whiskers). Statistical significance was assessed by Wilcoxon matched-pairs signed-rank test. (B) Neutralization of vesicular stomatitis virus-based pseudovirus particles harboring the indicated S proteins by donor-matched plasma (n = 37) taken before or after vaccination with the mRNA omicron JN.1 vaccine. Data represent GMT (colored columns) from a single experiment, conducted with 4 technical replicates. The lowest plasma dilution tested (dashed lines) and the assay threshold (lower limit of detection; gray shaded areas) are indicated. Information on response rates and mean fold change in neutralization after vaccination are indicated above the graphs. Statistical significance was assessed by Wilcoxon matched-pairs signed-rank test. Of note, for graphical reasons, plasma samples yielding a neutralization titer 50 (NT50) value <6.25 (limit of detection) were manually placed at the bottom of the axis. Individual neutralization data are available in the appendix (Fig S3). Abbreviations: GMT, geometric mean titer; IgG, immunoglobulin G; IQR, interquartile range; NA, not applicable; S, spike.

Next, we analyzed JN.1-reactive MBCs by means of flow cytometry (Figs S1 and S2; Tables S2 and S3). The proportion of SARS-CoV-2 S protein binding MBCs increased following JN.1 vaccination: MBC reacting exclusively with the receptor binding domain of JN.1 changed significantly and displayed a 3.9-fold increase 21 days post-vaccination (median of 0.11% MBCs before to a median of 0.43% MBCs after vaccination). Cross-reactive JN.1 receptor binding domain/Wuhan-Hu-1 S-binding MBCs and those only binding to Wuhan-Hu-1 S displayed a 2.3-fold and 1.8-fold change, respectively (Fig. 1A; Table S4; Figs S2 and Fig S3).

Third, we assessed changes in plasma neutralization on vaccination with the updated JN.1 omicron mRNA vaccine. For this, we used a vesicular stomatitis virus-based pseudovirus particle (pp) neutralization assay as published previously.^5^ We performed neutralization assays with patient plasma and used different pseudoviruses harboring S proteins of 6 distinct SARS-CoV-2 lineages (Fig 1B; Figs S1 and S4-S6). Before JN.1 vaccination, baseline response rates against tested variants were between 65% and 86% (86% for XBB.1.5_pp_, 78% for JN.1_pp_, 73% for KP.2_pp_, 65% for KP.2.3_pp_ and KP.3_pp_, and 68% for LB.1_pp_ [Fig 1B]). Particles bearing KP or LB.1 S sublineage proteins were less efficiently neutralized than JN.1-specific particles, supporting the notion of antibody evasion (Figs S4-S6). Twenty-one days after vaccination, plasma neutralization capacity for all pseudovirus types had increased significantly. We observed mean increases in neutralization between 8.3- and 23.5-fold, with a highly significant increase in JN.1-specific neutralization capacity of 18.7-fold (increase in plasma neutralization for the other SARS-CoV-2 lineages: XBB.1.5_pp_ 8.3-fold, KP.2_pp_ 22.5-fold, KP.2.3_pp_ 18.7-fold, KP.3_pp_ 25.5-fold, and LB.1_pp_ 23.5-fold; Fig 1B and Figs S4-S6).

Our study has some limitations. First, our data can only provide first insights into the immune response to the updated JN.1 vaccine in hemodialysis patients. Although our in vitro SARS-CoV-2 neutralization assay has been shown to serve as an adequate surrogate model for this purpose,^8^ further data are needed to assess immune trends, sustainability, and the clinical relevance of our findings. Furthermore, underlying diagnoses within our cohort of hemodialysis patients were diverse, which may have impacted individual immune responses. Also, it should be noted that, for MBC staining, we employed tetramer binding to the receptor binding domain of omicron JN.1 only. This is relevant when comparing changes in different antigen-specific MBC compartments to our previous study in hemodialysis patients after omicron XBB.1.5 vaccination^5^ in which we used tetramers reacting to the XBB.1.5 S protein. Further limitations can be found in Item S2 in the supplementary file.

In summary, our data provide the first evidence for a firm humoral immune response in dialysis patients after mRNA omicron JN.1 vaccination. This is important in the clinical care of this population, who are at particular risk for severe COVID-19^1^^,^^3^ and could benefit most from vaccination with updated COVID-19 vaccines. Continued adaptation of COVID-19 vaccines beyond, for example, XBB.1.5 vaccines to maintain protection is supported by extensive B cell receptor repertoire analysis^9^ but may be of clinical value only in populations at risk for severe COVID-19. The triple increase in MBCs reacting with the receptor binding domain of omicron JN.1 S protein combined with the strongly amplified neutralization against JN.1 and other contemporary omicron variants suggest that the updated mRNA omicron JN.1 vaccine could be highly effective at enhancing protection of dialysis patients and other vulnerable populations against evolving SARS-CoV-2 variants.

## Supplementary Materials

Supplementary File (PDF) Figure S1-S6; Item S1, S2; Table S1-S4.
