# Supplementary material for: Humoral Immune Responses in Dialysis Patients After mRNA Omicron JN.1 Vaccination
Source: Kidney Med. 2025 Jul 7;7(9):101067. doi: 10.1016/j.xkme.2025.101067 (PMC12408387; doi:10.1016/j.xkme.2025.101067)
Supplement: Supplementary File (PDF) — Figure S1-S6; Item S1, S2; Table S1-S4. [file mmc1.pdf]

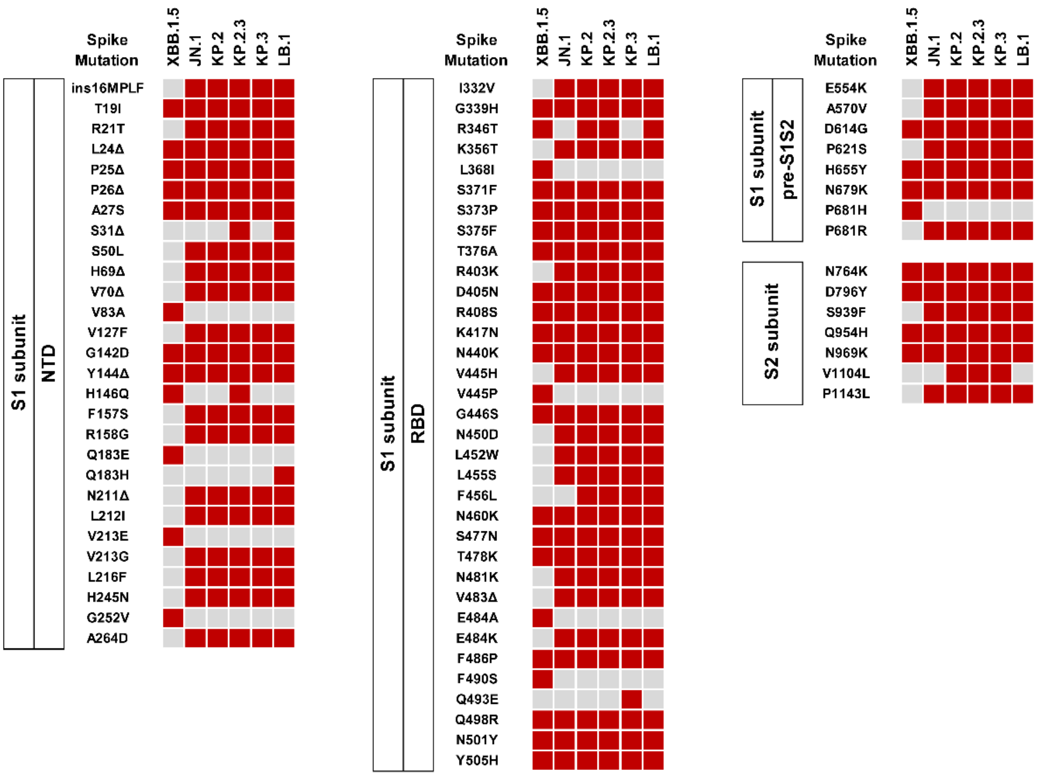

Fig. S1 | Overview of SARS-CoV-2 lineage-specific spike protein mutations

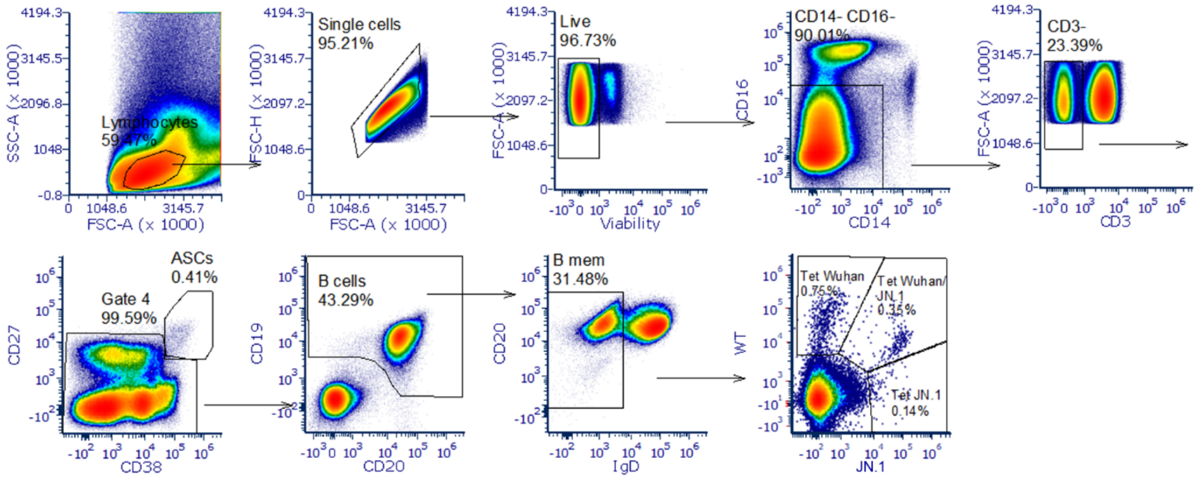

Fig. S2 | Gating strategy for SARS-CoV-2 S-reactive IgD⁻ cell populations in peripheral blood mononuclear cells. Tetramerised recombinant spike proteins from Wuhan Hu-1 (Tet Wuhan) or the receptor binding domain of spike omicron JN.1 (Tet JN.1) variants were used to identify memory B cells carrying B cell receptors binding to either one or both spike proteins. Pseudocolor plots show representative data from one individual. ASC, antibody secreting cells.

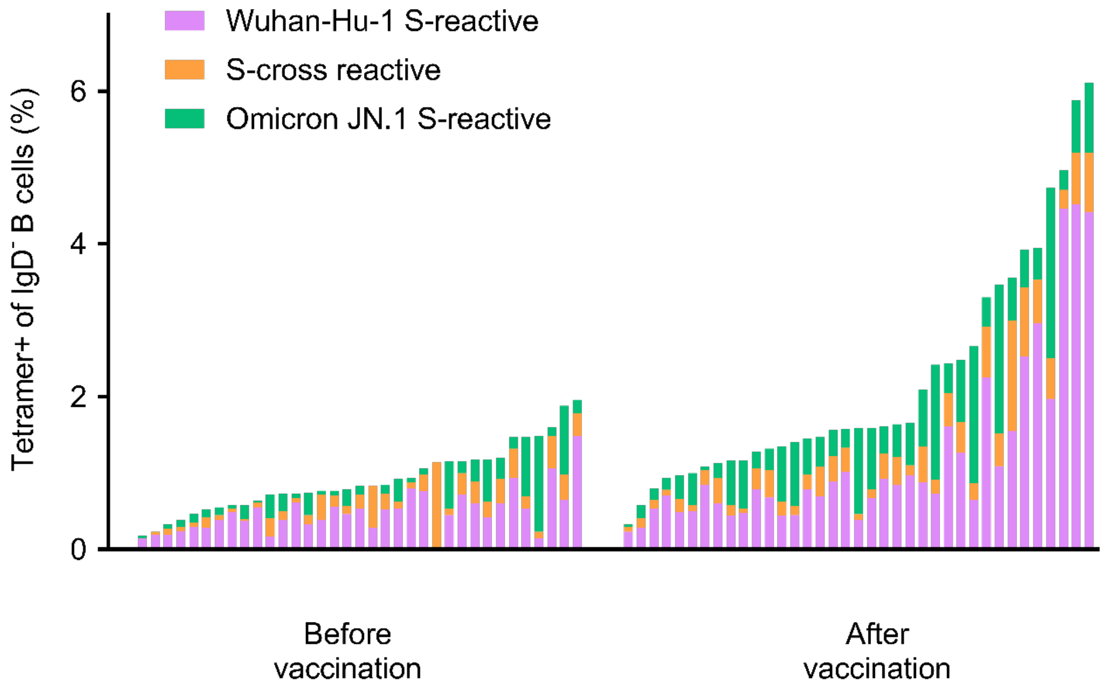

Fig. S3 | Individual data of changes in SARS-CoV-2 S-reactive IgD⁻ cell populations in peripheral blood mononuclear cells before and post mRNA omicron JN.1 vaccination. Tetramerised recombinant spike proteins from Wuhan-Hu-1 and the receptor binding domain of spike omicron JN.1 (Tet JN.1) variants were used to identify memory B cells carrying B cell receptors binding to either one or both spike proteins (S-cross reactive). B cells gated as shown in Fig. S2.

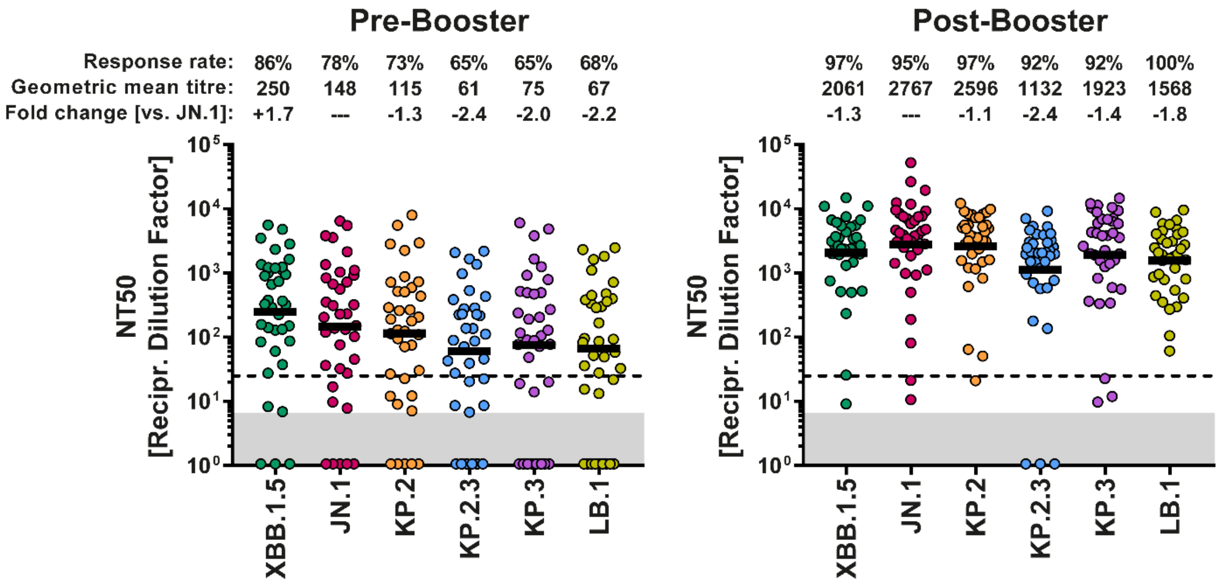

Fig. S4 | Humoral immune responses following mRNA omicron JN.1 vaccination. The data presented in this panel were regrouped from figure B to compare differences in SARS-CoV-2 lineage-specific neutralisation at baseline (before vaccination) and after vaccination. Information on GMT (also indicated by horizontal lines) response rates, and mean fold change in neutralisation compared with JN.1 pseudovirus particles are indicated above the graphs.

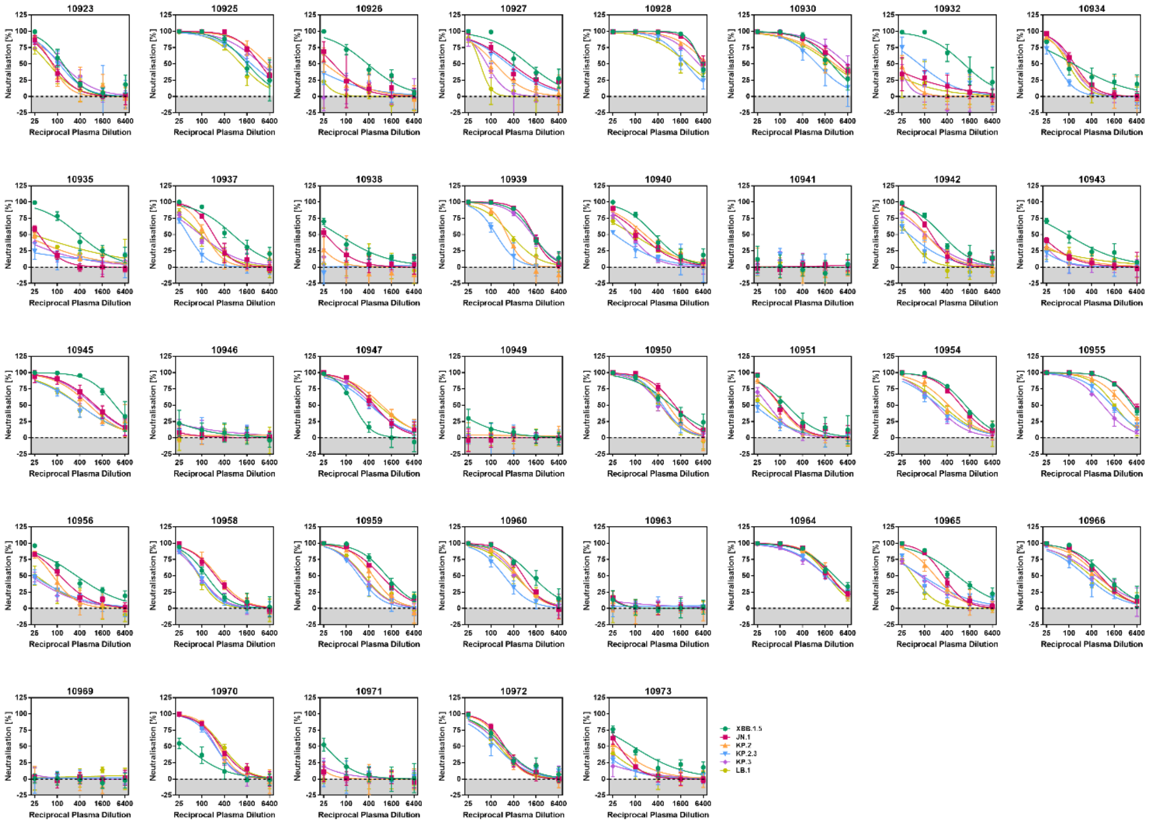

Fig. S5 | Individual neutralisation data for pre-vaccination plasma.

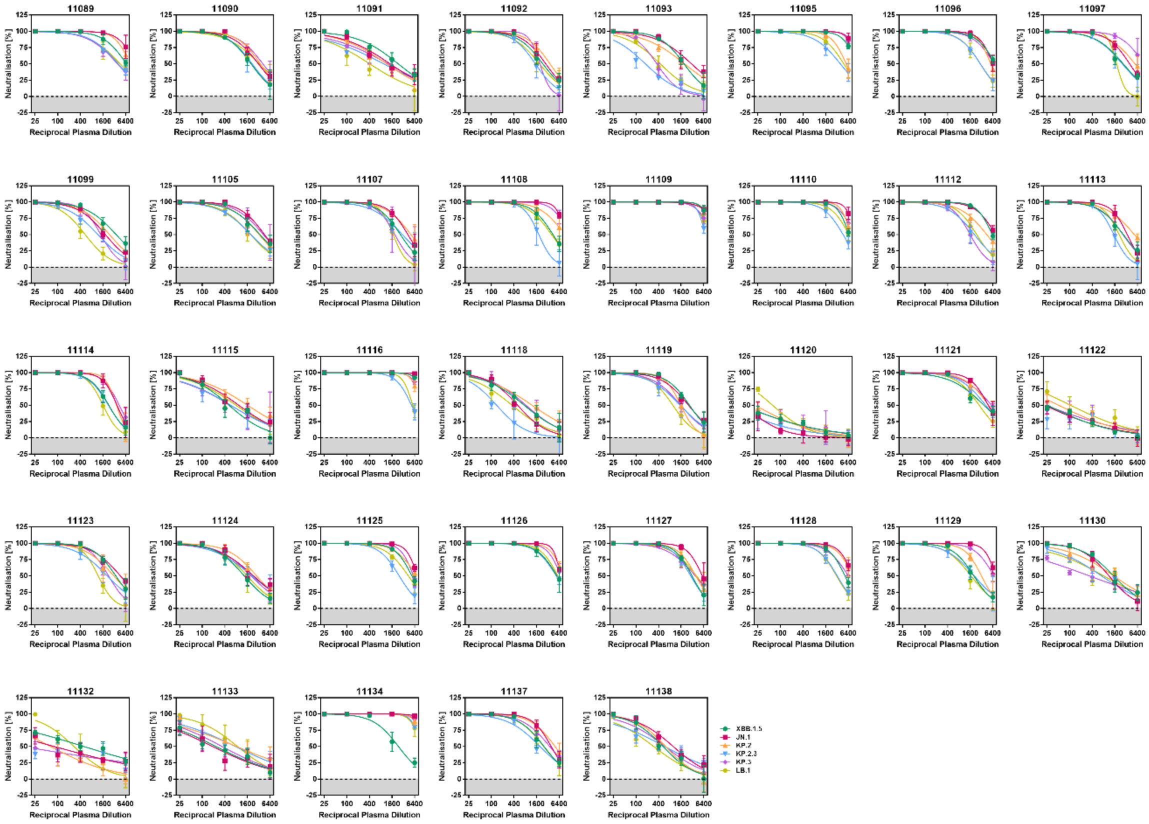

Fig. S6 | Individual neutralization data for post-vaccination plasma.

Item S1. Methods

Participants

Hemodialysis patients were recruited as part of the COVID-19 Contact (CoCo) Study (German Clinical Trial Registry, DRKS00021152). This is a prospective, observational study monitoring anti-SARS-CoV-2 immune responses in healthcare professionals and patients[1]. Initially, n=52 dialysis patients were immunized with 30µg of JN.1 Comirnaty® omicron/Bretovameran. Blood was drawn shortly before and 21 days after vaccination. Eight patients were excluded from the analysis due to confirmed SARS-CoV-2 infections during the observational period after vaccination, and n=7 patients were lost to follow up at day 21 due to death, hospitalization, or other (unspecified) reasons. We finally analyzed immune responses in n=37 dialysis patients. Details on the demographic data and case history of the included patients can be found in Suppl. Table 1.

Sample size calculations estimated that a sample size of n=43 should be sufficient for detection of a clinically relevant difference within the group, assuming that mean SARS-CoV-2 S1 protein-specific IgG levels of 2385 (SD=2394) BAU/ml double from pre-vaccination levels. Power calculation was performed using G*Power, Version 3.1.9.6. and based on anti-S IgG analyses in our previously published cohort of n=44 patients from previous recruitment phase of the CoCo dialysis study before XBB.1.5 vaccination[2], which is our best available estimate of pre-vaccination levels, correlation between groups 0.5. The estimate was based on a 2-tailed paired t-test of mean differences, with 98% power and 5% significance level. Based on our previous work, a loss-to follow-up rate of 20% was estimated. Following these calculations, we aimed at a sample size of n=52 vaccinated hemodialysis patients.

Serology

Serology was performed as previously described[1]. Briefly, we measured SARS-CoV-2 IgG by quantitative ELISA (Anti-SARS-CoV-2 QuantiVac-ELISA, EI 2606-9601-10G, and Anti-SARS-CoV-2 Omikron-ELISA, EI 2606-9601-30 G, both EUROIMMUN, Lübeck, Germany) according to the manufacturer's instructions (dilution up to 1:4,000). We used anti-S concentrations expressed as relative units (RU)/mL as assessed from a calibration curve with values above 11 RU/mL defined as positive. Values above the upper quantification limit of the assay (120 RU/mL for a 1:100 dilution) are set to 120 RU/mL (adapted to the used dilution) and used for further analysis. We provide results obtained with the QuantiVac ELISA in binding antibody units (BAU/mL), which were converted by multiplying RU/mL by 3.2, as specified by the manufacturer. We performed anti-SARS-CoV-2 nucleocapsid (NCP) IgG measurements according to the manufacturer's instructions (EUROIMMUN, Lübeck, Germany). We used an AESKU.READER (AESKU.GROUP, Wendelsheim, Germany) and the Gen5 2.01 Software for analysis.

Flow cytometric detection and analysis of SARS-CoV-2-specific B cells

For tetramer preparation, we used recombinant, biotinylated SARS-CoV-2 S protein (Wuhan-Hu-1 and omicron/JN.1) to detect SARS-CoV-2-spike-reactive B cells. Tetramerisation was performed as previously described[1]. Briefly, we tetramerised recombinant Wuhan-Hu-1 S proteins with fluorescently labelled streptavidin/R-phycoerythrin conjugate (Cat# S21388, ThermoFisher) and recombinant spike (RBD) omicron/JN.1 protein with fluorescently labelled streptavidin/ allophycocyanin (Cat# S868; ThermoFisher) [1]. We isolated fresh PBMCs samples, washed and re-suspended them in FACS buffer (PBS, 1 mg/mL BSA, 1 mmol/L EDTA) and stained cells with antibodies (Table S3) and tetramerised recombinant proteins against the Wuhan-Hu-1 S and omicron JN.1 S (RBD) for 20 min at room temperature. After two more wash steps, we acquired samples on a spectral flow cytometer (Cytek Northern Lights) and analyzed data using SpectroFlo and/or FCS Express software according to the gating strategy (Figure S2).

Production of vesicular stomatitis virus-based pseudovirus particles and pseudovirus neutralisation test (pVNT)

pVNTs were performed according to a previously published protocol[3]. In brief, 293T cells were transfected with S protein expression plasmid. Expression plasmids pCG1_SARS-2-SΔ18 XBB.1.5

(EPI_ISL_16239158; codon-optimised, deletion of last 18 aa residues at the C-terminus)[4] and pCG1_SARS-2-SΔ18 JN.1 (EPI_ISL_18530042; codon-optimised, deletion of the last 18 aa residues at the C-terminus)[5] have been described before. In addition, S protein expression plasmids pCG1_SARS-2-SΔ18 KP.2 (EPI_ISL_19197864; codon-optimised, deletion of the last 18 aa residues at the C-terminus), pCG1_SARS-2-SΔ18 KP.2.3 (EPI_ISL_19197559; codon-optimised, deletion of the last 18 aa residues at the C-terminus), pCG1_SARS-2-SΔ18 KP.3 (EPI_ISL_19203001; codon-optimised, deletion of last 18 aa residues at the C-terminus), and pCG1_SARS-2-SΔ18 LB.1 (EPI_ISL_19067004; codon-optimised, deletion of the last 18 aa residues at the C-terminus) were generated by introduction of the required mutations into plasmid pCG1_SARS-2-SΔ18 JN.1 (Figure S1). This was achieved by overlap-extension PCR using overlapping primers that harbour the respective mutations. At 24h posttransfection, the 293T cells were inoculated with a replication-deficient VSV vector that lacks the genetic information for the VSV glycoprotein and instead encodes for an enhanced green fluorescent protein and a firefly luciferase, VSV*ΔG-FLuc (kindly provided by Gert Zimmer, Institute of Virology and Immunology, Mittelhäusern, Switzerland)[6]. Following 1h of incubation at 37 °C and 5% CO_2, the cells were washed with PBS and further incubated with medium containing anti-VSV-G antibody (culture supernatant from I1-hybridoma cells; ATCC no. CRL-2700) to neutralise residual input virus. After 16-18h of incubation at 37 °C and 5% CO_2, the pseudovirus-containing supernatant was harvested, centrifuged (4,000 x g, 10 min) to remove cellular debris, and clarified supernatants were stored at -80 °C until further use.

For pVNTs, Vero76 cells (kindly provided by Andrea Maisner, Institute for Virology, Phillips University Marburg) were grown to confluence in 96-well plates. Next, pseudovirus particles were mixed with serial dilutions of heat-inactivated (56 °C, 30 min) plasma, incubated for 30 min at 37 °C, and finally inoculated onto the Vero cells in four technical replicates. Plasma dilutions were prepared in culture medium (final dilution range 1:25 to 1:6,400) and pseudovirus particles mixed with medium without plasma sample served as reference. At 16-18h postinoculation, pseudovirus infection was analyzed. For this, the culture supernatant was removed and cells were lysed with PBS containing 0.5 % Tergitol (Carl Roth; 30 min at room temperature). Thereafter, the cell lysates were transferred into white 96-well plates, mixed with luciferase substrate (Beetle-Juice, PJK) and incubated for 1 min, before luminescence was recorded using a Hidex Sense Microplate Reader Software (version 0.5.41.0). Efficiency of neutralisation was determined based on the relative inhibition of pseudovirus infection of Vero76 cells. Signals obtained for cells infected with pseudovirus particles incubated in the absence of plasma served as reference (no inhibition). Next, a non-linear regression model was used to calculate the neutralising titre 50 (NT50), which indicates the plasma dilution responsible for half-maximal inhibition. Of note, plasma samples that yielded NT50 values below 25 (lowest dilution tested) were defined as non-responders and samples that yielded NT50 values below 6.25 (limit of detection, LOD) were assigned an NT50 value of 3.125 (0.5 of LOD).

Statistics
GraphPad Prism 8.4 or 9.0 (GraphPad Software, USA) and SPSS 20.0.0 (IBM SPSS Statistics, USA) were employed for the statistical analyses. We included outliers into the analysis and excluded missing values pairwise. Mean (SD) was reported for normally distributed data, and median (IQR) for non-normally distributed values. For assessing differences within groups in non-normally distributed data, Wilcoxon matched-pairs signed rank test were employed. We transformed neutralisation titres to geometric mean titres before further analyzing this data.

Item S2. Limitations of the study and considerations for data interpretation

Our study has some limitations. As mentioned in the main body of our manuscript, our data can only provide first insights into the immune response to the updated JN.1 vaccine in an immunocompromised population. Further data is necessary to assess immune trends, sustainability, and clinical relevance of our findings. SARS-CoV-2 neutralisation was assessed by pVNT, which has been shown to serve as an adequate surrogate model for this purpose [6]. Post vaccination titres in some individuals were at the upper range of the dilutions used. Our findings await confirmation with clinical isolates and eventually validation in clinical studies. Pre-vaccination neutralisation and anti-S IgG responses in hemodialysis patients were lower than previously reported for health care-workers [7]. The tetramers used for MBC staining bind to the RBD of omicron JN.1 only, which – as mentioned in the main body of our research letter - is relevant when comparing changes in different antigen-specific MBC compartments to our previous study in hemodialysis patients after omicon XBB.1.5 vaccination[2], in which we used tetramers reacting to XBB.1.5 spike protein. Whilst the median time since last vaccination was similar and hemodialysis patients had received on average one more SARS-CoV-2 vaccination, the rate of hybrid immunity in these patients was 50% less, they were on average 20 years older, had more co-morbidities, and their blood was collected at day 21 (instead of day 13). We believe that these factors are likely influencing the magnitude of the post-vaccination response and limit direct comparisons. Whilst our study was powered to demonstrate clinically meaningful changes in immune surrogates after COVID-19 vaccination (anti-S IgG), our all sample size is small and limits interpretation beyond this parameter. The absence of a control group prevents direct comparisons with unvaccinated individuals or those receiving alternative vaccines, but there was no alternative vaccine available or recommended at the time of our study. The short follow-up period precludes conclusions on the durability of the immune response and clinical protection against breakthrough infections. All patients received the same dialysis modality, but heterogeneity in renal conditions or prior SARS-CoV-2 infections introduces potential confounding variables, making it difficult to isolate vaccine-induced effects from pre-existing immunity.

Item S3. Supplementary References

1. Stankov MV, Hoffmann M, Jauregui RG, et al. Humoral and cellular immune responses following BNT162b2 XBB.1.5 vaccination. medRxiv 2023: 2023.10.04.23296545.
2. Cossmann A, Hoffmann M, Stankov MV, et al. Immune responses following BNT162b2 XBB.1.5 vaccination in patients on haemodialysis in Germany. Lancet Infect Dis 2024; 24(3): e145-e6.
3. Arora P, Zhang L, Rocha C, et al. Comparable neutralisation evasion of SARS-CoV-2 omicron subvariants BA.1, BA.2, and BA.3. Lancet Infect Dis 2022; 22(6): 766-7.
4. Hoffmann M, Arora P, Nehlmeier I, et al. Profound neutralization evasion and augmented host cell entry are hallmarks of the fast-spreading SARS-CoV-2 lineage XBB.1.5. Cell Mol Immunol 2023; 20(4): 419-22.
5. Zhang L, Dopfer-Jablonka A, Cossmann A, et al. Rapid spread of the SARS-CoV-2 JN.1 lineage is associated with increased neutralization evasion. iScience 2024; 27(6): 109904.
6. Berger Rentsch M, Zimmer G. A vesicular stomatitis virus replicon-based bioassay for the rapid and sensitive determination of multi-species type I interferon. PLoS One 2011; 6(10): e25858.
7. Happle C, Hoffmann M, Kempf A, et al. Humoral immunity after mRNA SARS-CoV-2 omicron JN.1 vaccination. Lancet Infect Dis 2024.

Table S1. Demographics, vaccination and disease history of included dialysis patients

**Variable**	
Number of vaccinees	37
Age, Median (IQR), range years	68 (15), 28-90
Sex, male (%)	26 (70)
**Previous COVID-19 vaccinations and infections**	
Previous SARS-CoV-2 vaccination (%)	37 (100)
Previous SARS-CoV-2 omicron vaccination (%)	37 (100)
Median (range) months post last vaccination	11 (9-32)
6 vaccinations (%)	24 (64.9)
5 vaccinations (%)	7 (18.9)
≤4 vaccinations (%)	6 (16.2)
Previous SARS-CoV-2 infections (%)	16 (43)
Previous SARS-CoV-2 omicron infection (%)	16 (43)
Median (range) months post last infection	24 (15-31)
**Clinical history**	
Median months since start of hemodialysis (IQR), range	53 (65), 4-289
Using immunosuppressive medication (%)*	6 (16)
Obesity with BMI >30 n= (%)	11 (30)
Diabetes mellitus (%)	12 (32)
Cardiovascular disease (%)	23 (62)
**Underlying renal disease**	
Diabetic nephropathy	8 (22)
Hypertensive kidney disease	7 (19)
IgA nephropathy	3 (8)
Other diseases	19 (51)
**Baseline laboratory assessment**	
Albumin, median (range) g/L	40 (29-46)
Transferrin, median (range) mg/L	183 (127-275)
C reactive protein, median (range) mg/L	2.7 (0.6-21.5)

*see Methods for further details

Table S2. Tetramer Antigen Conjugate

Antigen	Conjugate	Clone	Order no.	Company	Dilution
SARS-CoV-2 Spike Trimer (Wuhan Hu-1)	Biotinylated	NA	#SPN-C82E9	Acrobiosystems	100
SARS-CoV-2 Spike Trimer (Omicron RBD JN.1)	Biotinylated	NA	#0BB1D-F001	Acrobiosystems	400

Table S3. Antibody panel

Antigen	Conjugate	Clone	Order no.	Company	Dilution
CD16	Alexa Fluor 488	3G8	302019	BioLegend	100
CD14	PE/Cy5	M5E2	301864	BioLegend	100
CD20	Brilliant Violet 570	2H7	302332	BioLegend	100
CD38	APC-Fire810	HIT2	303550	BioLegend	100
CD3	Alexa Fluor 532	UCHT1	58-0038-42	Invitrogen	100
CD27	Alexa Fluor 700	O323	56-0279-42	Invitrogen	100
CD19	Pacific Blue	SJ25C1	363036	BioLegend	100
IgD	BV480	1A6-2	566138	BD Horizon	100
Viability	Zombie NIR	NA	423106	BioLegend	1000

Table. S4 | Anti-S IgG and anti-S reactive MBC pre and post mRNA JN.1 vaccination immune responses following mRNA omicron JN.1 vaccination.

	Median	IQR
Pre-Vac Wuhan-Hu-1 S-specific IgG [BAU/mL]	1817	2670.9
Post-Vac Wuhan-Hu-1 S-specific IgG [BAU/mL]	5413	9024
Pre-Vac Omicron S-specific IgG [RU/mL]	170	266.1
Post-Vac Omicron S-specific IgG [RU/mL]	796	758.8
Pre-Vac Wuhan-Hu-1 S-reactive MBC (%)	0.45	0.34
Post-Vac Wuhan-Hu-1 S-reactive MBC (%)	0.79	0.90
Pre-Vac cross-reactive MBC (%)	0.12	0.23
Post-Vac cross-reactive MBC (%)	0.27	0.31
Pre-Vac omicron JN.1 RBD-reactive MBC (%)	0.11	0.23
Post-Vac omicron JN.1 RBD-reactive MBC (%)	0.43	0.50
